# The Impact of Exclosure Duration on Plant Species Diversity in a Desert Grassland and the Relative Contribution of Plant Groups

**DOI:** 10.1002/ece3.70698

**Published:** 2024-12-23

**Authors:** Jiaojiao Huang, Shijie Lv, Hongmei Liu, Shengyun Ma

**Affiliations:** ^1^ College of Science Inner Mongolia Agricultural University Hohhot Inner Mongolia China; ^2^ Forestry Research Institute of Inner Mongolia Autonomous Region Hohhot Inner Mongolia China

**Keywords:** desert steppes, exclosure, α diversity, β diversity, γ diversity

## Abstract

Plant species diversity has long been a focal point in ecological studies. In order to study the changes in species diversity at different spatial scales (α, β, and γ diversities) in the restoration process of grassland vegetation in fragile desert steps, this study took desert steppe of Inner Mongolia as the research object and employed a two‐factor experimental design that combined exclosure years (the years when an area was isolated to prevent grazing and other disturbances) with years of monitoring (the years when data were collected). It analyzed the plant groups (dominant species, common species, and rare species) and species diversity, and obtained the preliminary conclusions as follows: The optimal exclosure duration for promoting species diversity balance in desert steppe management is between 16 and 18 years. Short‐term exclosure enhances species diversity by promoting recovery in overgrazed systems, while long‐term exclosure may reduce diversity due to dominant species proliferation and inhibited regeneration. Increasing the duration of exclosure (the period from the initial exclosure year to the year of monitoring) can improve plant species diversity. Exclosure years and years of monitoring exhibited a significantly positive influence on α, β, and γ diversities, with a negative interaction effect between exclosure years and years of monitoring. In addition, plant groups played a significant role in diversity at different spatial scales. Contribution to α diversity ranked as follows: rare species > common species > dominant species; contribution to β diversity ranked as rare species > dominant species > common species; contribution to γ diversity ranked as common species > dominant species > rare species. Rare species played a crucial role in maintaining diversity stability within the community and diminishing gradient differences, and common species were instrumental in upholding landscape features.

## Graphical abstract

This study focused on plant species diversity in the restoration of grassland vegetation in desert steppes. Using a two‐factor experimental design in Inner Mongolia's desert steppe, the research found that enclosure and monitoring years positively influenced α, β, and γ diversities, with a negative interaction effect between them. Plant groups had a significant role on diversity. Rare species are crucial for maintaining community stability and reducing gradient differences, and common species play an important role in upholding landscape features.

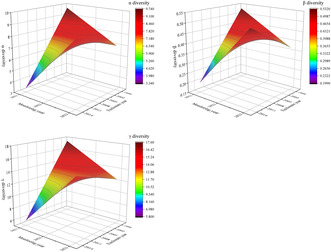

## 1 Introduction

The well‐being of grassland ecosystems, integral to the global ecosystem, is intricately linked to biodiversity, ecological balance, and human welfare (Hector and Bagchi 2007; Kang et al. 2007). In recent years, global environmental concerns have arisen due to grassland degradation caused by factors such as overgrazing (Liang et al. 2009; Schönbach et al. 2011) and climate change (Wang et al. 2007). In response, targeted restoration measures, including exclosure (Golodets, Kigel, and Sternberg 2010; Shrestha and Stahl 2008), seasonal fallow grazing (Katoh et al. 1998), replanting (Feng et al. 2010), and grassland fertilization (Wang et al. 2020), are gradually being implemented in degraded grasslands. Exclosure refers to the practice of fencing off or isolating an area to prevent grazing and other disturbances by large herbivores (Aerts, Nyssen, and Haile, 2008). It is characterized by low investment, high energy efficiency, and ease of implementation (Bendz 1988; Cong et al. 2021; Teketay et al. 2018). The impact of grazing exclosure on grassland species diversity has consistently been the focus of academic inquiry. Some studies have indicated that as the duration of exclosure increases, there is an upward trend in plant species diversity (Abebe et al. 2006; Mengistu et al. 2005; Pei, Fu, and Wan 2008). Research on alpine meadow steppe in the Qinghai‐Tibet Plateau revealed that compared to free‐grazing grasslands, exclosure led to increased community cover and productivity but decreased species diversity (Chen et al. 2021). Other studies suggested that a 14‐year exclosure period is most conducive to the restoration of degraded grasslands. During this time, community density and cover continue to increase to their maximum before gradually decreasing (Shan et al. 2008). Hence, it is believed that short‐term exclosure benefits the regeneration and reproduction of grassland communities (Shashemene 2014), while an excessively prolonged closure period may harm the grassland (Chen et al. 2023; Sun et al. 2020). All these findings underscore that exclosure can effectively enhance the growth of grassland vegetation, with its impact varying over time.

The effects of exclosure on plant diversity are related to spatial scale (Tang and Fang 2004). Biodiversity can be divided into α, β, and γ diversities based on different spatial scales (Whittaker 1960), which are both related and different. α diversity pertains to the diversity of species within a specific community or habitat, while γ diversity relates to the diversity of species across a range of habitats within a given region. β diversity, acting as a bridge between α and γ diversity, refers to the rate and extent of change in species diversity along an environmental gradient from one community or habitat to another (Anderson et al. 2011). Exclosures effectively alleviate grazing pressure. According to Tilman's (1982) resource competition theory, when the intensity of competition for limited resources is reduced, species can more efficiently allocate and utilize available resources. This enhanced resource partitioning can promote niche differentiation, ultimately leading to an increase in α diversity. At a medium scale, exclosures may alter the dominance of certain plant species, leading to changes in plant community structure and composition. This aligns with the environmental gradient theory, which emphasizes how variations in spatial conditions drive β diversity (Whittaker 1960; Anderson, Ellingsen, and McArdle 2006). At the landscape scale, exclosure typically involves the strategic use of smaller, scattered fenced areas to study changes in ecosystem structure and function, ultimately affecting plant species diversity.

Exclosure measures can effectively enhance the ecological environment of the grassland. However, variations in grassland degradation may arise due to differing exclosure methods (such as seasonal and year‐round exclosure) and exclosure durations (the period from the initial exclosure year to the year of monitoring). Prior studies on the impact of exclosure duration on plant species diversity have predominantly focused on α diversity, with limited attention to β and γ diversities, and the exclosure duration only considered the exclosure years and ignored the years of monitoring. Due to the differences between different years of monitoring, the study of the effects of the duration of exclosure on species diversity only by exclosure years will lead to a certain bias into the results. For instance, a year of monitoring with favorable hydrothermal conditions (e.g., adequate rainfall and moderate temperatures) might exhibit higher species diversity compared to a year with drought or extreme temperatures. Additionally, in grassland ecosystems, the ecological status and significance of plant groups (dominant species, common species, and rare species) vary. Dominant species garner considerable attention due to their predominant role in grasslands and their significant impact on community species diversity and ecosystem functionality. However, common and rare species are often overlooked. Common species have a widespread distribution and play a crucial role in maintaining community stability, while rare species, despite their smaller population sizes, may also have significant impacts on ecosystem dynamics under specific environmental conditions.

In summary, this study focuses on the Inner Mongolia desert steppe and utilizes a two‐factor experimental design incorporating years of monitoring and exclosure years to accurately assess the impact of exclosure duration on species diversity. Additionally, it explores whether the role of plant groups (dominant species, common species, and rare species) in influencing species diversity remains consistent under exclosure conditions. The study seeks to address the following questions: (1) the response of species diversity to the exclosure years (the years when an area was isolated to prevent grazing and other disturbances) and the years of monitoring (the years when data were collected to assess the ecological impacts of exclosure); (2) the variation characteristics and patterns of species diversity at different spatial scales (α, β, and γ); (3) whether the effects of plant groups on diversity at different spatial scales (α, β, and γ diversities) are consistent. The resolution of these inquiries not only clarifies the impacts of exclosure on species diversity across various spatial scales but also establishes a scientific foundation for grassland vegetation restoration and ecological protection.

## 2 Materials and Methods

### 2.1 Natural Overview of the Study Area

**FIGURE 1:**
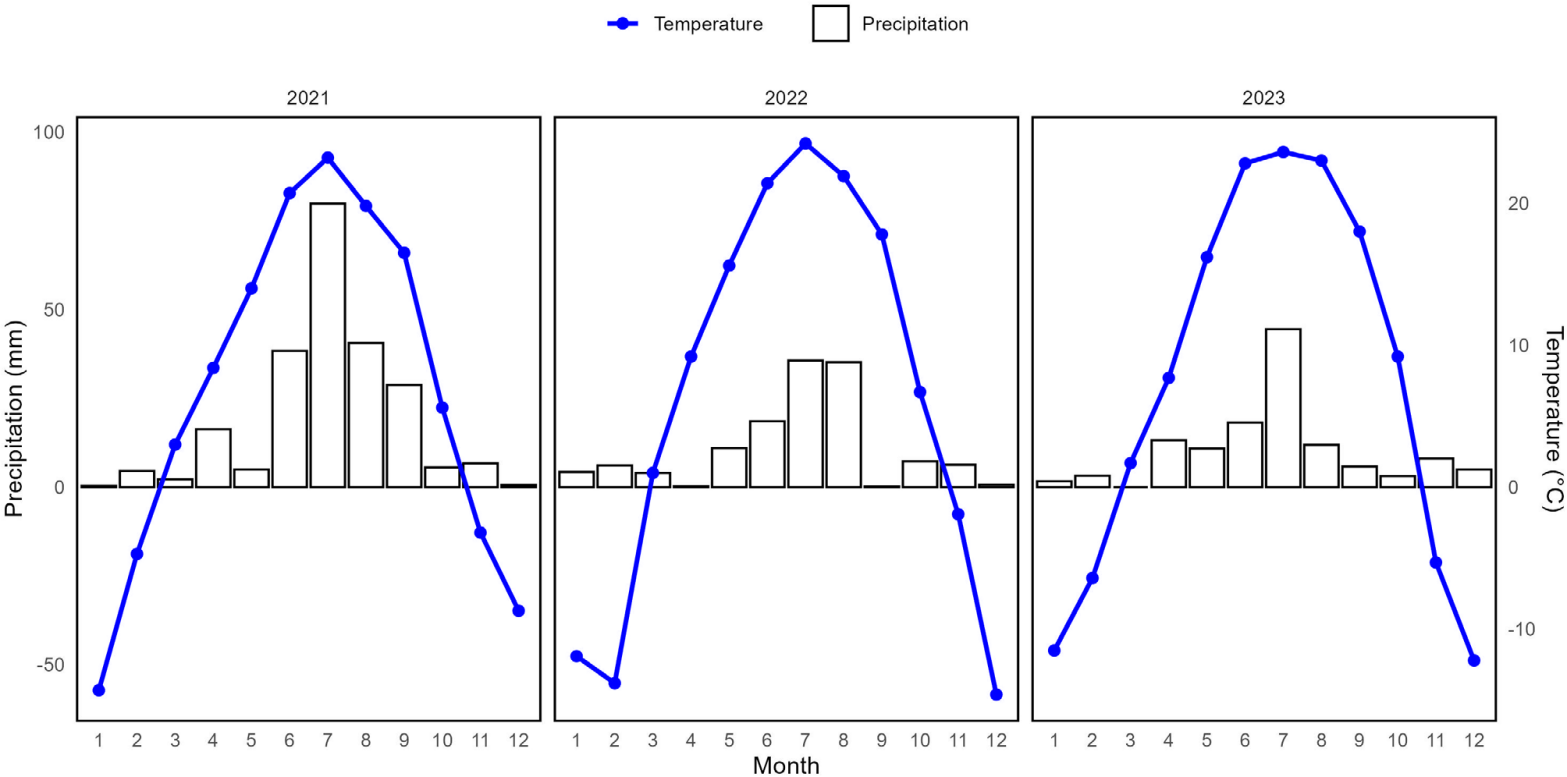
Precipitation and temperature in the study area for years of monitoring 2021–2023.

The study area is located in the central region of the Inner Mongolia Autonomous Region, specifically in the western part of Xilingol League, Sunit Right Banner (41°55′–43°39′ N,111°08′–114°16′ E). Situated in the transition zone between typical grassland and desert, this region has a unique geographical location and harsh habitat conditions, making it highly sensitive to human disturbances and climate change (Yang et al. 2019). (Before the establishment of the exclosures, this land experienced heavy grazing practices, which led to significant pressure on the vegetation and soil health. Following the installation of the exclosures, all grazing activities ceased.) The terrain varies in elevation from 1000 to 1400 m. The climate is classified as temperate semi‐arid continental monsoon climate with dryness, abundant sunshine, strong winds, scant rainfall, and significant temperature variations between day and night. The temperature ranges from a low of −38.8°C to a high of 38.7°C, with an average of 5.9°C. The area experiences a frost‐free period of 210 days and receives an annual average precipitation of 203.5 mm. Total annual sunshine is approximately 129 days. Prevailing northwest winds average 4.5 m/s. The annual precipitation for the years of monitoring 2021 to 2023 was 229.10, 129.70, and 125.60 mm, respectively. The corresponding average annual temperatures were 6.69°C, 6.30°C, and 7.23°C (Figure 1). The vegetation in the study area primarily comprises desert steppe with clusters of *Stipa brevifloris*. Throughout the monitoring period from 2021 to 2023, a total of 28 plant species, comprising 13 families and 25 genera, were identified. This included 6 annual and biennial herbs, 19 perennial herbs, and 3 shrubs. Families exhibiting a high species diversity include Poaceae (Gramineae) (7 species), Asteraceae (4 species), Amaryllidaceae (4 species), and Fabaceae (Leguminosae) (3 species), with monospecific families constituting 61.5% of the total family count. Based on the vegetation types ( *Stipa breviflora* + *Allium polyrhizum* + *Cleistogenes songorica*) in the study area and the importance value (IV) data obtained from 52 sampled plots, plant species were categorized into three community membership types: dominant species, common species, and rare species (Liu 2007; Pan et al. 2018; Yang et al. 2014) (Table 1). Dominant species encompassed *Allium polyrhizum*, *Cleistogenes songorica*, and *Stipa breviflora*. Common species (IV > 1) included nine species such as *Convolvulus ammannii*, *Neopallasia pectinata*, and *Eragrostis minor* . Meanwhile, rare species (IV < 1) encompassed 15 species like *Allium ramosum* , *Pappophorum brachystachyum* , and *Scorzonera muriculata*.

**TABLE 1:** Classification of dominant species, common species, and rare species in the study area.

Classification of species	Species name	Importance value (%)
Dominant species	*Cleistogenes songorica*	21.39
	*Allium polyrhizum*	17.24
	*Stipa breviflora*	10.03
Common species	*Convolvulus ammannii*	11.08
	*Neopallasia pectinata*	11.55
	*Eragrostis minor*	6.79
	*Caragana stenophylla*	5.43
	*Carex tristachya*	3.82
	*Salsola collina*	2.47
	*Bassia prostrata*	2.46
	*Allium tenuissimum*	1.69
	*Setaria viridis*	1.22
	*Artemisia scoparia*	1.20
Rare species	*Allium ramosum*	0.70
	*Pappophorum brachystachyum*	0.47
	*Scorzonera muriculata*	0.44
	*Aster altaicus*	0.25
	*Tragus mongolorum*	0.25
	*Erodium stephanianum*	0.24
	*Asparagus gobicus*	0.24
	*Tribulus terrestris*	0.23
	*Lappula squarrosa*	0.17
	*Allium mongolicum*	0.16
	*Chloris virgata*	0.16
	*Parthenocissus tricuspidata*	0.14
	*Astragalus galactites*	0.08
	*Gueldenstaedtia verna*	0.08
	*Lagochilus ilicifolius*	0.04

### 2.2 Experimental Design

The grassland plots enclosed in 1999 (enclosed area: 1 ha, equivalent to 10,000 m^2^), 2005 (enclosed area: 2.57 ha, equivalent to 25,700 m^2^), and 2014 (enclosed area: 2.57 ha, equivalent to 25,700 m^2^) were selected as research sites. From August 2021 to August 2023, five 1 m × 1 m plots were randomly chosen within each enclosed plot each year. Additionally, in August 2021, seven extra 1 m × 1 m plots were randomly selected within the 1999 enclosed plot, making a total of 52 plots (15 plots (2021) + 15 plots (2022) + 15 plots (2023) + 7 plots (additional 2021) = 52 plots). Plant species, height (utilizing a graduated ruler, the height of three of the tallest plants of a particular species within each plot was measured. The average height was then recorded), cover (a grid‐based visual estimation method (Qin et al. 2006) was employed, dividing each 1 m × 1 m plot into 100 equal‐sized grid cells. The number of grid cells covered by each species was visually estimated), and density (the number of plant clusters of each species present within each plot) within each plot were recorded for every species present.

### 2.3 Plant Species Diversity and Important Value Calculation

In this paper, the plant species diversity index calculated based on frequency data was used to explore the effects of different exclosure years (1999, 2005, and 2014) and years of monitoring (2021, 2022, and 2023) on α, β, and γ diversities, and the plant diversity and importance values were calculated as follows:

1. α diversity index: α = number of species in each 1 m^2^ survey plot;
2. β diversity index: $\beta =1-\bar{\alpha }/\gamma$ (Tuomisto 2010) where $\bar{\alpha }$ is the mean value of the α diversity index for the duration of exclosure;
3. γ diversity index: γ = total number of species recorded under each exclosure year;
4. Importance Value (*IV*): *IV* = (relative height + relative cover + relative density)/3 × 100%.
  relative height = average height of a species/sum of average heights of all species;
  relative cover = cover of a species/total cover of all species;
  relative density = number of clusters of a species/total number of clusters of all species.
5. The duration of exclosure = year of monitoring—exclosure years

### 2.4 Statistical Analysis

To address the challenge of unequal sampling sizes across different years of monitoring, we utilized version 2.6.6 of the vegan package within R 4.3.0 to perform sample rarefaction (Gotelli and Colwell 2001), ensuring consistency and comparability in our data. Then to better reflect the impact of the exclosure duration and maintain consistency across variables, we designated 2014 as the reference year and assigned it a value of 1. By establishing this reference point, we standardized exclosure years relative to 2014. 2005, 9 years prior to 2014, was represented as 10, indicating a 10‐year exclosure duration. 1999, 15 years prior to 2014, was represented as 16, signifying a 16‐year exclosure duration. Similarly, the 3 years of monitoring of 2021, 2022, and 2023 were replaced by 1, 2, and 3. Then, the exclosure years, years of monitoring, and their interaction terms were taken as independent variables, and α, β, and γ diversities are taken as dependent variables, respectively, and the lm function in R 4.3.0 was used to make a general linear model to analyze the effects of exclosure years and years of monitoring on α, β, and γ diversities. The results were presented by plotting point‐whisker plots using the ggcoefstat function of the ggstatsplot 0.12.1 package. Secondly, based on the fitted equations, data simulation was performed using Excel 2016's VBA (Visual Basic for Applications). Calculations were conducted at 0.2‐year intervals for both years of monitoring and exclosure years, resulting in 10,200 combinations. The simulation results were presented by Origin 2024 software. Finally, using the lmer function in the lme4 1.1.35.1 package of the R 4.3.0, a mixed linear model was built with plant groups as a fixed effect and year of monitoring and year of exclosure as random effects. Then, the total variation in α (β, γ) diversity was further divided into three components: dominant species, common species, and rare species, and with the aid of the mixed linear model, the contribution of different plant groups to α, β, and γ diversities was analyzed using the glmm.hp. function of the glmm.hp. 0.1.0 package.

## 3 Results and Analysis

### 3.1 Effects of Exclosure Years and Years of Monitoring on Diversity

**FIGURE 2:**
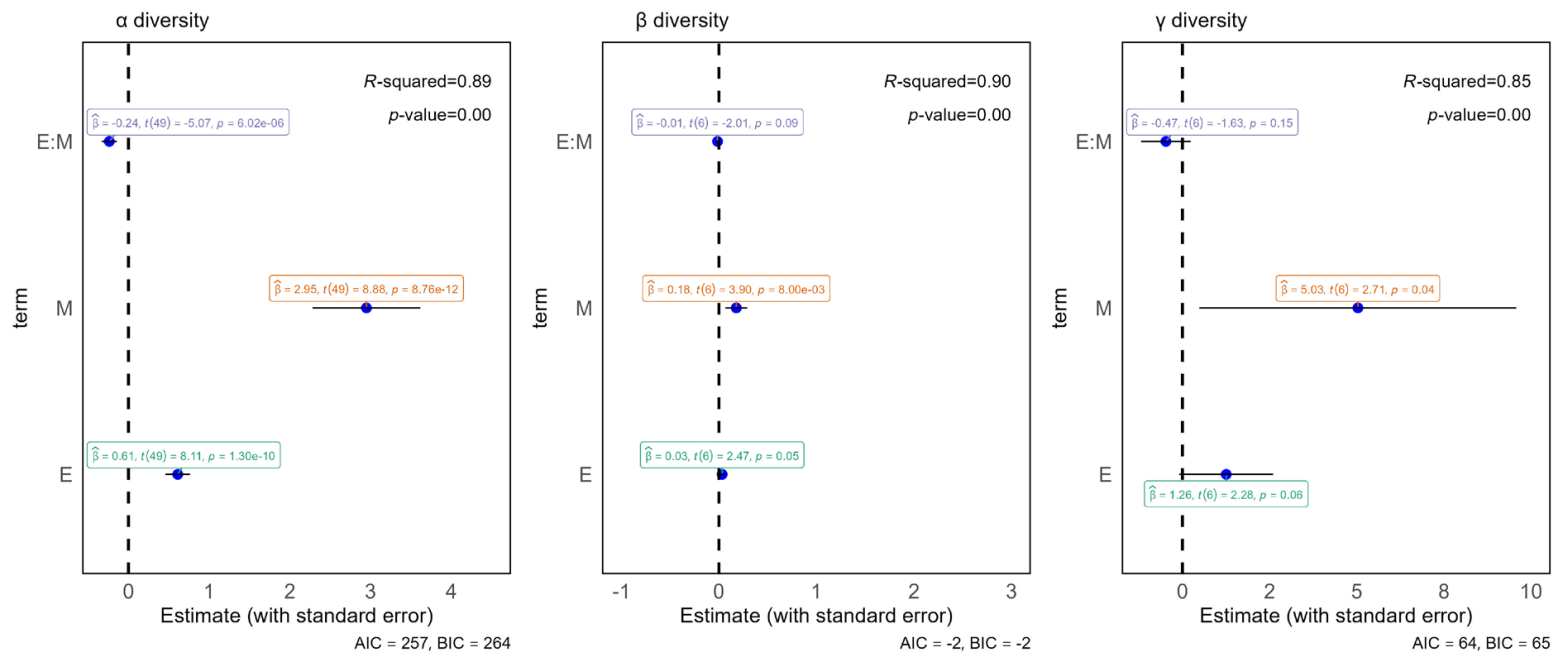
Point‐whisker plots of the effects of exclosure year and year of monitoring and their interaction terms on α, β, and γ diversities (point estimates of regression coefficients are shown as points, and confidence intervals as whiskers). Exclosure.year:Monitoring.year denotes the interaction term; *R*‐squared and *p*‐value are the overall goodness‐of‐fit and *p*‐value of each regression model, respectively; $\hat{\beta }$ denotes the coefficient estimate of the respective variable, *t* denotes the *t* value of the respective variable with degrees of freedom, and *p* denotes the *p*‐value of the respective variable; AIC and BIC stand for the Akaike information criterion and the Bayesian information criterion.

The regression results of α, β, and γ diversities reveal that the models fit well, with *R* ^2^ values of 0.89 (*p* < 0.01), 0.90 (*p* < 0.01), and 0.85 (*p* < 0.01), respectively. This indicates that a three models as a whole reaches the significance level (α: AIC = 257, BIC = 264; β: AIC = −2, BIC = −2; γ: AIC = 64, BIC = 65) (Figure 2). For α diversity, the interaction between exclosure year and the year of monitoring has a significant negative effect (coefficient = −0.24, *p* < 0.01), meaning that α diversity decreases when exclosure years are earlier (closer to 1999) and the year of monitoring is later (closer to 2023). Conversely, diversity increases when the year of monitoring is closer to 2021. Both the exclosure year (coefficient = 0.61, *p* < 0.01) and the year of monitoring (coefficient = 2.95, *p* < 0.01) individually show significant positive effects on α diversity, indicating their strong contribution to diversity when exclosure year is held constant. For β diversity, the interaction term shows a small negative effect (coefficient = −0.01, *p* > 0.05), while the exclosure year (coefficient = 0.03, *p* < 0.05) and the year of monitoring (coefficient = 0.18, *p* < 0.01) both significantly increase diversity. For γ diversity, the interaction term (coefficient = −0.47, *p* > 0.05) and exclosure year (coefficient = 1.26, *p* > 0.05) both have non‐significant effects, indicating limited interaction between these factors on gamma diversity. However, the year of monitoring (coefficient = 5.03, *p* < 0.05) has a strong positive impact, suggesting that species richness at the larger community scale increases substantially over the year of monitoring.

### 3.2 Variation of α, β, and γ Diversities Across Exclosure Years and Years of Monitoring

**FIGURE 3:**
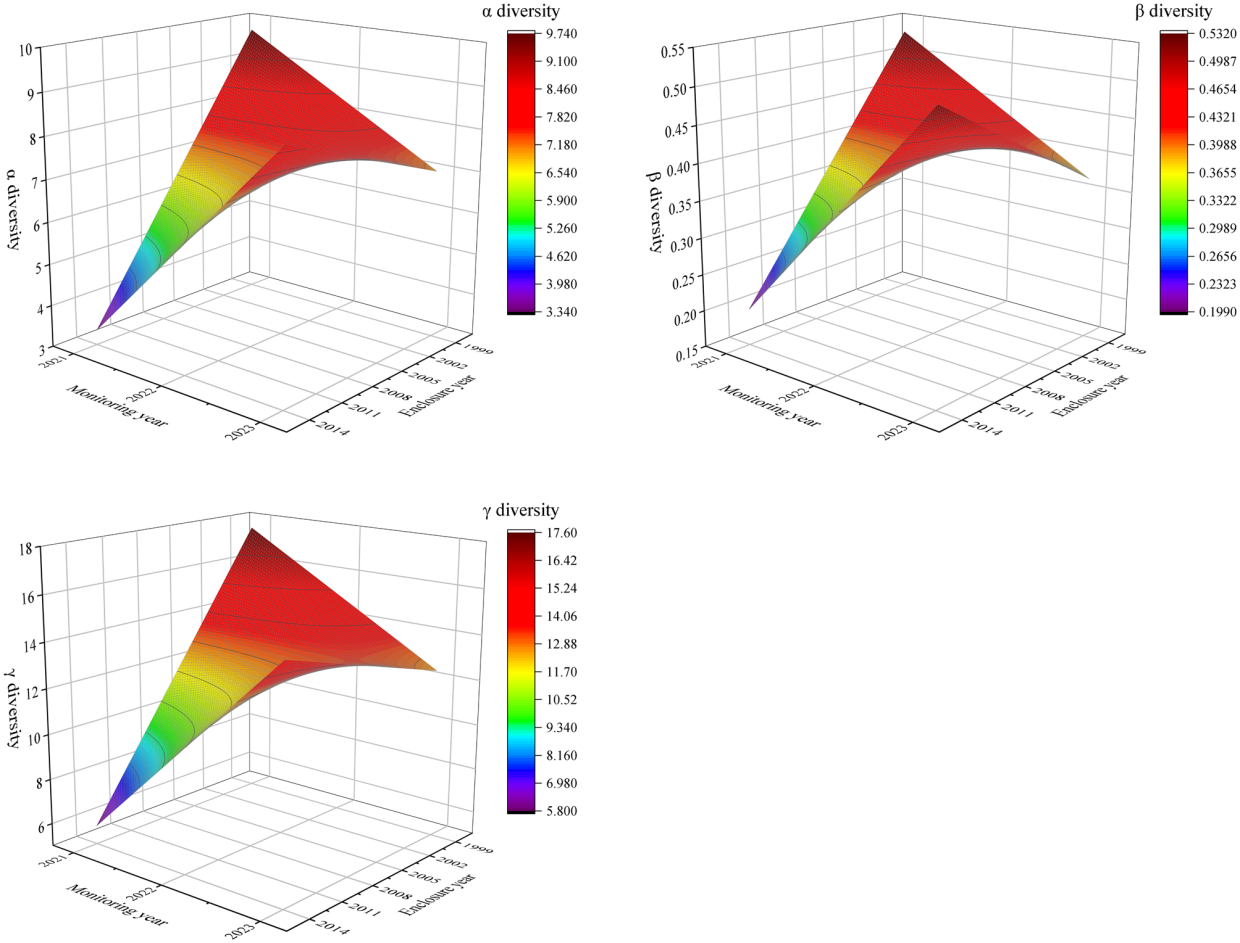
Results of α, β, and γ diversity simulations based on regression equations. The color changes from purple to red to indicate the gradual increase in the diversity of α (β, γ).

α, β, and γ diversities were simulated using the fitted equations obtained from the regression, and it is found that the α, β, and γ diversities show a “saddle” shape under the influence of the year of exclosure and the year of monitoring (Figure 3). Specifically, the grassland community exhibits the minimum α, β, and γ diversities when the exclosure year is 2014, and the year of monitoring is 2021. Subsequently, as the year of monitoring progresses to 2022 and 2023, the duration of exclosure for the grassland community gradually increases, leading to a more rapid ascent in α, β, and γ diversities to higher levels. Conversely, during the exclosure year of 1999, as the year of monitoring regresses from 2023 to 2021, the duration of exclosure for the grassland community progressively shortened, resulting in a swifter increase in α, β, and γ diversities. The α, β, and γ diversities all reach their maximum when the year of monitoring is 2021.

**FIGURE 4:**
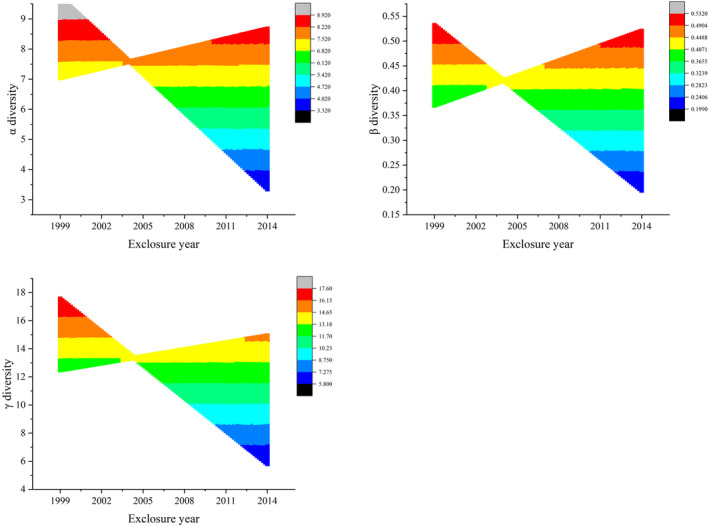
Results obtained by projecting backward the plane where the *y*–*z* axis is located in Figure 3. The figure reveals different levels of fluctuation in α, β, and γ diversities around 2005. Before and after 2005, diversity levels change with varying years of monitoring, but around the exclosure year 2005, species diversity remains at a stable level. This indicates that the exclosure year 2005 is optimal for maintaining species diversity and ecosystem health.

The simulated data are compared with the original data to find the theoretical optimal values of α, β, and γ diversities (Table 2). It can be seen that the theoretical optimal value of α diversity is 9.39, and the best exclosure year is approximately 1999. From the perspective of the year of monitoring, the optimum year of monitoring aligns with approximately 2021. Regarding β diversity, the theoretical optimal value is 0.52, indicating the optimal range for exclosure years spans from 2002 to 2003, while the optimal years of monitoring fall within the range of 2021 to 2022. As for γ diversity, the theoretical optimal value is 14.94, suggesting the optimal exclosure year is roughly 1999, and the optimal year of monitoring corresponds to approximately 2022. Projecting the years of exclosure onto the plane of α, β, and γ diversities in Figure 3 reveals that the optimal exclosure year is around 2005 (Figure 4). During this period, α, β, and γ diversity values are stable and balanced. In contrast, shorter exclosure durations (7–9 years) show increasing diversity, while longer exclosure durations (over 22 years) show a decline in diversity. Therefore, exclosure year around 2005 provides the optimal balance for maintaining species diversity.

**TABLE 2:** Results of α, β, and γ diversity simulation optimization.

	Year of monitoring	Exclosure year	Optimal value
α diversity	2021	1999	9.39
β diversity	2021–2022	2002–2003	0.52
γ diversity	2022	1999	14.94

### 3.3 Effects of Plant Groups on α, β, and γ Diversities

The outcomes of the mixed linear model reveal a significant impact of plant groups on α, β, and γ diversities (*p* < 0.01) (Table 3). The total variation in α (β and γ) diversity was further decomposed into three components: dominant species, common species, and rare species, allowing for an analysis of the magnitude of the contribution made by each plant groups. Concerning α diversity, rare species make the most substantial contribution, followed by dominant species and, lastly, common species (Table 4). In terms of β diversity, dominant species have the greatest impact, followed by rare species and, lastly, common species. Similarly, for γ diversity, common species make the most significant contribution, followed by rare species and, finally, dominant species.

**TABLE 3:** Effects of plant groups on α, β, and γ diversities.

Response variable	Main effect	*F*	*p*
α diversity	Plant groups	80.03	0.00***
β diversity	Plant groups	103.03	0.00***
γ diversity	Plant groups	17.75	0.00***

*** *p* < 0.01.

**TABLE 4:** Contributions of different plant groups to α, β, and γ diversities (%).

	Dominant species	Common species	Rare species
α diversity	8.81	40.88	50.31
β diversity	45.72	8.19	46.09
γ diversity	21.96	58.68	19.36

## 4 Discussion

### 4.1 Optimal Exclosure Duration for Desert Steppe Grassland Management

Exclosure represents an extreme approach to grassland management, exhibiting both protective and destructive effects on the ecological environment (Xu et al. 2020). The key to effective grassland management is determining the exclosure duration most conducive to vegetation recovery while judiciously balancing the proportions of “rest” and “use” (Li et al. 2013). Research by Liu et al. demonstrates that species diversity tends to increase initially and then decrease with extended exclosure durations. Short‐term exclosure can significantly enhance species diversity, whereas long‐term exclosure may diminish it, which aligns with the findings of our study (Liu et al. 2023; Wu et al. 2009).

Based on our findings, short‐term exclosure (7–9 years) is optimal for rapidly improving grassland conditions, especially in ecosystems that have previously experienced heavy grazing pressure. During this phase, the absence of grazing allows for substantial vegetation recovery, particularly of rare and sensitive species that might not survive under continuous grazing pressure. Short‐term exclosures also reduce competition between species, providing an ideal window for diversity to peak. During medium‐term exclosure (16–18 years), represents a period of equilibrium where species diversity stabilizes. At this stage, resource availability and habitat conditions peak, supporting a diverse array of species and forming a stable and diverse grassland ecosystem. Importantly, long‐term exclosure (over 22 years) can lead to a decline in species diversity. This decline may result from the over‐proliferation of dominant species, which increases competition for resources. And accumulated dead leaves on the surface can obstruct sunlight, water, and nutrients from penetrating the soil, inhibiting plant regeneration and reproduction, and thus reducing species diversity (Liu et al. 2019). In summary, our findings suggest that the optimal exclosure duration for desert steppe management is 16–18 years for maintaining species diversity balance. Short‐term exclosure enhances rapid vegetation recovery, while medium‐term exclosure promotes ecosystem stability. Exclosures exceeding 22 years may require active management to avoid negative effects such as reduced biodiversity. Moreover, determining the optimal duration of exclosure should also consider factors such as the degree of grassland degradation (Teng et al. 2020), seasonal variations (Angassa and Oba 2010), and exclosure methods (Yang et al. 2022). These factors significantly influence species diversity and ecosystem health.

### 4.2 Differential Contributions of Plant Groups to α, β, and γ Diversities

The α, β, and γ diversities were significantly influenced by plant groups, with distinct contributions from different plant categories. Rare species emerged as pivotal contributors to α diversity, surpassing common and dominant species. This prominence arises from the ability of rare species to respond rapidly to specific environmental conditions (Wamelink, Goedhart, and Frissel 2014), which can allow them to adapt successfully when such conditions align with their ecological requirements. Consequently, under favorable environmental changes, rare species can thrive in local habitats. Their occupation of specific ecological niches further distinguishes them from other species. This allows rare species to manifest high α diversity in local environments. In contrast, exclosure tends to induce substantial overlap between common and dominant species within ecological niches, escalating competition for resources in the same area (Slobodchikoff and Schulz 1980). This process leads to the formation of a relatively homogeneous ecological community, thereby diminishing their contribution to α diversity.

Concerning β diversity, rare species exert the greatest influence, succeeded by dominant species, with common species contributing the least. This is because the majority of species in communities are rare. Rare species typically inhabit specific microenvironments, leading to a greater diversity of species composition among different sites, thereby increasing β diversity. Dominant species, characterized by broad ecological niche occupancy (Grime 1998), high abundance, and strong competitiveness, maintain consistent species compositions across diverse gradients. Thus, they contribute relatively less to β diversity. Common species exhibit relatively stable population dynamics among different sites. Their widespread distribution leads to less pronounced differences in species composition among different habitats, resulting in the smallest contribution to β diversity. Regarding γ diversity, common species play more substantial roles, while dominant and rare species contribute less. Common species often possess weed‐like characteristics, such as rapid growth and wide adaptability. They also have disturbance resistance and serve as bridges between different ecosystems, making significant contributions to overall γ diversity and ecosystem stability. Rare species, although enhancing γ diversity by their presence, exhibit lower relative abundance in ecosystems, limiting their overall contribution (James and Rathbun 1981). Dominant species, by successfully occupying a wide range of ecological niches, reduce opportunities for other species within the same niches, thereby impacting γ diversity.

## 5 Conclusion

There is a negative interaction effect between exclosure years and years of monitoring, and the optimal exclosure period falls within the range of 16–18 years. Plant groups exert significant effects on α, β, and γ diversities, though these effects vary. In terms of α diversity, its contribution was as follows: rare species, common species, and dominant species; in terms of β diversity, its contribution was from large to small: rare species, dominant species, and common species; in terms of γ diversity, its contribution was from large to small: common species, dominant species, and rare species. It can be seen that rare species play a critical role in maintaining the stability of diversity within the community and mitigating gradient differences within the ecosystem; common species emerge as essential contributors to maintaining landscape characteristics.

## Author Contributions

**Jiaojiao Huang:** conceptualization (equal), investigation (equal), writing – original draft (equal), writing – review and editing (equal). **Shijie Lv:** formal analysis (equal), investigation (equal). **Hongmei Liu:** funding acquisition (equal), investigation (equal), resources (equal). **Shengyun Ma:** investigation (equal), supervision (equal).

## Conflicts of Interest

The authors declare no conflicts of interest.

## Supporting information


Data S1

Data S2

## Data Availability

The data that support the findings of this study are available in the Supporting Information of this article.
