# Supplementary material for: The Impact of Exclosure Duration on Plant Species Diversity in a Desert Grassland and the Relative Contribution of Plant Groups
Source: Ecol Evol. 2024 Dec 23;14(12):e70698. doi: 10.1002/ece3.70698 (PMC11664206; doi:10.1002/ece3.70698)
Supplement: Supplementary file 1 — Data S1 [file ECE3-14-e70698-s001.doc]

**Prepare**

library(Matrix)

library(lme4)

library(ggplot2)

library(lmerTest)

library(ggstatsplot)

library(glmm.hp)

library(patchwork)

Sys.setlocale("LC_ALL","Chinese")

**Effects of enclosure years and monitoring years on diversity (general linear model)**

my<-read.csv("C:\\Users\\admin\\Desktop\\regression.csv")

head(my)

p<-lm(α.diversity~Enclosure.year*Monitoring.year+0,data=my)

summary(p)

p1<-ggcoefstats(p,title = "α diversity")

p2<-p1 + annotate("text", x = 4.5, y = 3.6, label = "R-squared=0.9129", hjust = 1, vjust = 1)

p3<-p2 + annotate("text", x = 4.5, y = 3.4, label = "p-value=0.0014", hjust = 1, vjust = 1)

p4<-p3 + theme(panel.grid=element_blank())

p4

ggsave(p4,filename="α diversity.png",path="C:\\Users\\admin\\Desktop\\figure",dpi=600)

pp<-lm(β.diversity~Enclosure.year*Monitoring.year+0,data=my)

summary(pp)

p<-ggcoefstats(pp,title="β diversity")

p

p2<-p + annotate("text", x = 0.32, y = 3.6, label = "R-squared=0.9105", hjust = 1, vjust = 1)

p3<-p2 + annotate("text", x = 0.32, y = 3.4, label = "p-value=0.0015", hjust = 1, vjust = 1)

p41<-p3 + theme(panel.grid=element_blank())

p41

ggsave(p41,filename="β diversity.png",path="C:\\Users\\admin\\Desktop\\figure",dpi=600)

pp1<-lm(γ.diversity~Enclosure.year*Monitoring.year+0,data=my)

summary(pp1)

p1<-ggcoefstats(pp1,title = "γ diversity")

p1

p2<-p1 + annotate("text", x = 11.5, y = 3.6, label = "R-squared=0.8631", hjust = 1, vjust = 1)

p3<-p2 + annotate("text", x = 11.5, y = 3.4, label = "p-value=0.0053", hjust = 1, vjust = 1)

p42<-p3 + theme(panel.grid=element_blank())

p42

ggsave(p42,filename="γ diversity",path="C:\\Users\\admin\\Desktop\\figure",dpi=600)

pz<-p4+p41+p42+plot_layout(ncol = 3)

pz

ggsave(pz,filename="diversity.png",path="C:\\Users\\admin\\Desktop\\figure",dpi=600)

**Effects of plant groups on α, β and γ diversity (mixed linear model)**

my1<-read.csv("C:\\Users\\admin\\Desktop\\regression2.csv")

head(my1)

n1<-lmer(α.diversity~classify+(1|Monitoring.year)+(1|Enclosure.year),data=my1)

summary(n1)

anova(n1)

n2<-lmer(β.diversity~classify+(1|Monitoring.year)+(1|Enclosure.year),data=my1)

summary(n2)

anova(n2)

n3<-lmer(γ.diversity~classify+(1|Monitoring.year)+(1|Enclosure.year),data=my1)

summary(n3)

anova(n3)

**Contribute**

u1<-lmer(α.diversity~dominant+common+rare+(1|Monitoring.year)+(1|Enclosure.year),data=my1)

summary(u1)

anova(u1)

glmm.hp(u1)

u2<-lmer(β.diversity~dominant+common+rare+(1|Monitoring.year)+(1|Enclosure.year),data=my1)

summary(u2)

anova(u2)

glmm.hp(u2)

u3<-lmer(γ.diversity~dominant+common+rare+(1|Monitoring.year)+(1|Enclosure.year),data=my1)

summary(u3)

anova(u3)

glmm.hp(u3)
